# Venetoclax in the treatment of secondary plasma cell leukemia with translocation t(11;14): a case report and literature review

**DOI:** 10.3389/fonc.2024.1390747

**Published:** 2024-07-10

**Authors:** Hesham Elsabah, Rola Ghasoub, Halima El Omri, Maria Benkhadra, Honar Cherif, Ruba Y. Taha

**Affiliations:** ^1^ Department of Hematology and Bone Marrow Transplant, National Center for Cancer Care and Research (NCCCR), Hamad Medical Corporation (HMC), Doha, Qatar; ^2^ Pharmacy Department, National Center for Cancer Care and Research (NCCCR), Hamad Medical Corporation (HMC), Doha, Qatar

**Keywords:** plasma cell leukemia, BCL-2 inhibitor, venetoclax, t(11;14) plasma cell neoplasm, multiple myeloma

## Abstract

**Introduction:**

Venetoclax is a BCL-2 inhibitor with proven efficacy in patients with multiple myeloma (MM) and translocation t(11;14). However, its role in plasma cell leukemia (PCL) remains unclear. Herein, we aimed to report a case of relapsed MM with secondary PCL and t(11;14) achieving complete (CR) and durable remission with venetoclax therapy.

**Case presentation:**

A 52-year-old gentleman was diagnosed with MM-free light chain lambda (ISS III) in December 2016. He received induction therapy, followed by autologous stem cell transplant. (ASCT) in May 2017 and maintenance. A year later, the patient relapsed with secondary PCL. His cytogenetics analysis revealed t(11; 14). The patient failed salvage chemotherapy and was shifted to venetoclax with dexamethasone treatment. The patient attained complete remission (CR), which was maintained for two years and a half before he developed fatal COVID-19 pneumonia.

**Conclusion:**

In comparison with the reported literature, this case report offers the latest compilation of the available evidence on the use of venetoclax in patients with PCL. Furthermore, our patient achieved CR for the longest reported durable response in literature thus far. Prospective clinical trials are needed to elucidate the optimal dosage, combination, and duration of treatment, ensuring better representation and generalizability of the findings. Meanwhile, venetoclax may be considered as a therapeutic option in patients with PCL t(11;14).

## Introduction

Plasma cell leukemia (PCL) is a rare and aggressive form of plasma cell neoplasm that is characterized by the uncontrollable proliferation of malignant plasma cells in the bone marrow and peripheral blood (1). It represents approximately 2% of all plasma cell neoplasms, making it a challenging clinical entity to manage (1). The diagnostic criteria were recently revised from 20% to ≥5% of circulating plasma cells in peripheral blood smears. PCL can be primary, where the initial presentation is at diagnosis, or secondary to preexisting multiple myeloma (MM) (2, 3). Considering the rarity of the disease, there is a limited number of recruitable patients for controlled studies (2). This poses a challenge to the management of PCL despite the emergence of novel therapies (2).

Venetoclax is a BCL-2 inhibitor that has demonstrated its efficacy in multiple studies in patients with MM with t(11,14) (4). Given the high prevalence (30–50%) of the t(11,14) in the PCL population, BCL-2 inhibition may be considered a therapeutic target in this population (5). However, only a few case reports have discussed the use of venetoclax in PCL. Herein, we report a case of relapsed MM with secondary PCL with t(11;14) responding to treatment with the oral selective BCL-2 inhibitor venetoclax and achieving complete and durable remission. In addition, we have included a summary of the available literature on the use of venetoclax in PCL patients.

## Case report

A 52-year-old gentleman presented in December 2016 with anemia, hypercalcemia, a destructive left iliac bone lesion, and a past medical history that was significant for renal agenesis, hypothyroidism, type 2 diabetes mellitus (DM), and peripheral neuropathy. His laboratory tests revealed white blood cell (WBC) 6.9 x 10^3/uL, hemoglobin (Hb) 9.2 gm/dL, platelet 353 x 10^3/u, calcium (Ca) 2.8 mmol/L, creatinine (Cr) 130 ummol/L, albumin 43 g/L and beta- 2 microglobulin level of (B2M) 6.44 mg/L.Bone marrow (BM) biopsy was hypercellular (~60-80% cellularity) with extensive infiltration with plasma cell (> 90%). Flow cytometry analysis revealed monotypic plasma cells (approximately 60%) with cytoplasmic Lambda light chain expression. Multiple myeloma workup was positive for a high lambda free light chain (LaFLC) level of 2,289 mg/L, normal Kappa free light chain (KaFLC) of 10.7 mg/mL, low KaFLC to LaFLC ratio of 0.004, and monoclonal band on serum protein electrophoresis (SPE) that proved to be LaFLC upon immunofixation. He was, hence, diagnosed with MM-free light chain lambda (ISS III).

Following his diagnosis with MM he received induction therapy with VRd (bortezomib, lenalidomide and dexamethasone) for 5 cycles. This was followed by High Dose Melphalan-Autologous Stem Cell Transplantation (HDM-ASCT) in May 2017. The patient achieved a partial response (PR) at day 100 post -ASCT. He received subsequent local radiotherapy as 46 Gy in 23 fractions to the left iliac bone between October and November 2017. He was maintained on lenalidomide with dexamethasone between December 2017 and August 2018.

In late July 2018, the patient started having progressive pancytopenia: WBC 0.7 x 10^3/uL, neutrophil 0.3 x 10^3/uL, Hb 8.7 gm/dL, and platelet 43 x 10^3/uL. In September 2018, samples from peripheral blood and BM aspiration were infiltrated with 37% and 93% plasma cells, respectively. Flow cytometry BM showed an abnormal population of monotypic plasma cells comprises 19%, with cytoplasmic lambda light chain restriction and aberrant expression of CD56 and CD117. This confirmed a disease relapse with extensive BM infiltration and secondary PCL. The cytogenetic analysis revealed t(11; 14). Subsequently, the patient was started on D-PACE (dexamethasone, cyclophosphamide, doxorubicin, etoposide, cisplatin) in combination with Carfilzomib (20 mg/m2 days 1- 2; 27 mg/m2 on days 8-9, 15-16) and daratumumab (16 mg/kg weekly IV).The disease evaluation after one cycle in November 2018 revealed disease progression with extensive BM plasma cell infiltration (around 90% infiltration by plasma cells).

Following disease progression, chemotherapy was stopped, and the patient was started on venetoclax 400 mg daily, which was subsequently increased to 800 mg in combination with weekly dexamethasone 20 mg. The percentage of circulating plasma cells in the peripheral blood was 37% before venetoclax initiation. Subsequently, the WBC dropped, and the number of circulating plasma cells was very low for detection by manual differential testing.

The follow-up BM biopsy 21 days after the initiation of venetoclax revealed 2% plasma cell infiltration. At 4 months post-therapy, the patient maintained complete remission (CR) with 1% plasma cells and normal cytogenetics. The treatment course was complicated with mild diarrhea and uncontrolled DM.

**Figure 1:**
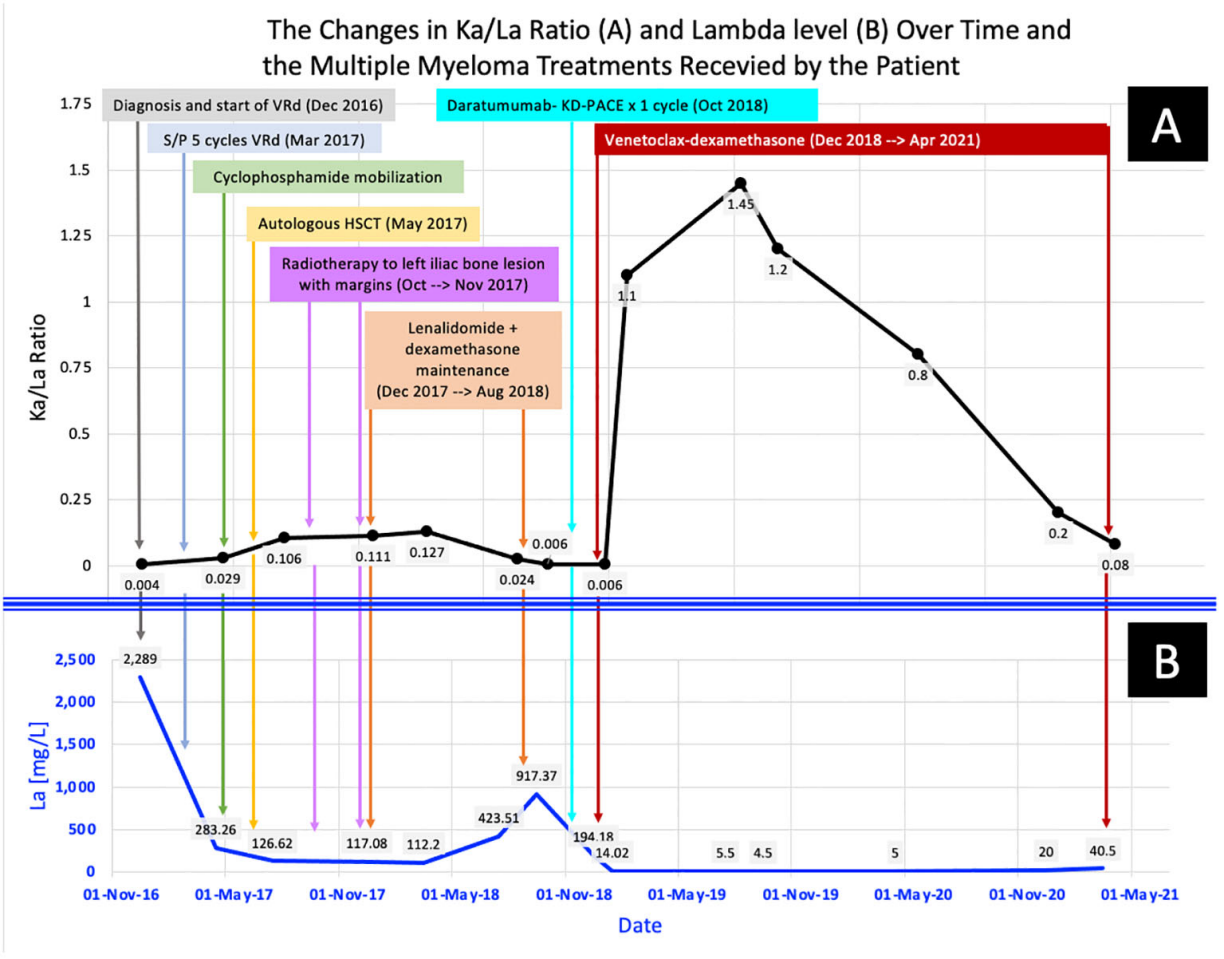
The changes in the patient’s multiple myeloma parameters across time: from diagnosis to the latest disease evaluation (before the fatal COVID-19 Infection). **(A)** shows the changes in KaFLC/LaFLC ratio, while **(B)** shows the changes in LaFLC levels.

The patient remained in CR until May 2021, when he developed severe COVID-19 pneumonia and passed away due to COVID-19-related complications. **Figures 1A, B** show the changes in KaFLC to LaFLC ratio and LaFLC level, respectively from diagnosis to latest disease evaluation (before the COVID-19 infection) and the therapy received at each time point.

## Literature review and discussion

PCL as a rare plasma cell neoplasm, lacks the standardization of a gold standard therapeutic approach. However, prior literature has demonstrated the superiority of the use of drug combinations involving novel agents (immunomodulators and proteasome inhibitors) in PCL in terms of responses and overall survival (6, 7). Despite combination therapies, the prognosis of PCL remains poor, with the median survival of patients with primary PCL ranging between 7 and 14 months (8). This is considered better than that of secondary PCL for which the median survival ranges between 2 and 7 months (8). This poor prognosis further added to the need for newer and more effective therapies for PCL treatment.

Venetoclax, a novel targeted therapy, has emerged as a promising therapeutic option for patients with PCL. However, there is limited data on the use of venetoclax in PCL with no existing prospective clinical trial or treatment protocol. Szita et al., described real- world evidence on the use of venetoclax in patients with plasma cell neoplasms and t(11;14) (9). They reported the results of six patients with PCL of whom two were diagnosed with primary PCL, while the other four were diagnosed with secondary PCL (9). All six patients responded to venetoclax therapy (one with PR, four with very good PR, and one with CR) (9). Unfortunately, all patients with secondary PCL eventually passed away with a median progression-free survival (PFS) of 10 months and an overall survival (OS) of 12.2 months (9). Moreover, Cotte et al., retrospectively described eight cases, from Monter Cancer Center and Northwell Health Cancer Institute, in which half of the cases had secondary PCL (10). Only one case was treated with venetoclax and had an overall survival of 5 months (10).

In terms of case reports, only thirteen patient cases have been published in the literature. Of the published cases, nine patients had primary PCL, while four patients had secondary PCL (4–6, 11–20). All patients harbored t(11;14) or BCL 2 overexpression; 3 patients had 17p deletion or TP53 mutation (6, 11, 19); and three cases had a complex karyotype (14, 15, 18). Venetoclax was given in combination in almost all cases except three patients (5, 12, 15) with a dose range of 100 mg to 1200 mg. In the case reported by Mikala et al., pharmacological enhancement was used to achieve an equivalent dose of 1200 mg daily while administering 400 mg of venetoclax through the concomitant use of a CYP3A4 inhibitor (20). **Table 1** summarizes the cases published on the use of venetoclax in patients with PCL.

**Table 1:** Summary of the published plasma cell leukemia cases with BCL-2 overexpression that incorporated venetoclax in the management pProtocol.

Publication	Age	M/F	Plasma cell neoplasm	Cytogenetics	Previous lines of treatment	Details of venetoclax	Reported response	Duration of response with venetoclax
**Jelinek et al. (2018)** (5)	58	M	pPCL relapse IgG lambda	t(11;14) 9:14	1. Vd x 2 cycles 2. HyperCVAD-VD 3. ASCT	Venetoclax 1200 mg in 21-day cycles	CR MRD negative	Last follow-up was at 7 months, no data for after publication
**Gonsalves et al. (2017)** (6)	55	F	pPCL relapse kappa light chain	t(11;14) 17p del	1. KRd x 4 cycles 2. VDT-PACE + ASCT 3. Daratumumab, doxorubicin, cyclophosphamide, dexamethasone	Venetoclax 800mg + bortezomib + daratumumab + dexamethasone	MRD negative	Case reported at 5 months from venetoclax initiation
**Asnawi et al. (2023)** (11)	59	F	pPCL IgG lambda	t(11;14) 17p del	VCd x 1 cycle	VCd + Venetoclax 100 mg x 5 cycles followed by D-VRD x 1 cycle with Al-HSCT	CR	18 months at time of publication
**Glavey et al. (2020)** (12)	73	F	sPCL IgG kappa MM	t(11;14)	Refractory to bortezomib, carfilzomib, lenalidomide, ixazomib	Venetoclax 50 mg - 400 mg	VGPR	Case reported at 9 months from venetoclax initiation
**VO et al. (2022)** (13)	64	M	pPCL Relapse	t(11;14)	1. HyperCD 2. D-KRd	Venetoclax 400 mg + dexamethasone 4 mg weekly	CR for 9 months	9 months
**Abu Zaanona et al. (2021)** (14)	70	M	sPCL IgG MM	t(11;14) Monosomy 13 Trisomy 3,9,15 1q duplication	KRd + ASCT	Venetoclax 100 mg + DVD x 4 cycles	Refractory	NA, he progressed during cycles 3 and 4
**Roy et al. (2022)** (15)	64	M	pPCL free light chain kappa	t(11;14)	1. Vd-PACE 2. KPD-PAVE 3. KPVd+ tandem ASCT	Induction: 400 mg for 14 days Then ➔ Venetoclax 100 – 200 mg	PR	Patient progressed after 4 months of treatment
**Roy et al. (2022)** (15)	40	F	pPCL IgG lambda	-13 Hypodiploidy Gain 1q21 14:20	N/A	KCD-PAVE + Venetoclax 800 mg D1-10 then ASCT and maintenance venetoclax 400 mg + Ixazomib	PR	2 cycles followed by maintenance venetoclax Ixazomib (duration not specified)
**Kupsh et al. (2019)** (16)	67	M	sPCL IgG kappa MM	t(11;14)	N/A	Venetoclax, bortezomib, dexamethasone	VGPR	Not reported
**Yang et al. (2021)** (17)	57	F	Relapse pPCL free light chain lambda	t(11;14)	1. VTCD 2. VRCD + Rd/VRd alternation maintenance 3. IRCD 4. BCMA/CAR-T 5. DRD 6. Selinexor/DEX	Venetoclax 300 mg + chidamide + dexamethasone	VGPR	7 months at time of publication
**Villiani et al. (2020)** (18)	58	M	Refractory pPCL IgM	t(11;14) Complex KT{TP53 del	1. Vd-PACE 2. Hyper-CAD + Carfilzomib 3. Daratumumab + lenalidomide+ Cyclophosphamide + dexamethasone	Venetoclax 400mg + dexamethasone 40 mg weekly	VGPR	15 months at time of publication
**Nalghranyan et al. (2019)** (19)	74	M	Refractory pPCL	t(11;14) Biallelic TP53 del17p	VDT-PACE	Venetoclax 300 mg + Daratumumab + dexamethasone	sCR MRD negative	10 months at time of publication
**Mikala et al. (2018)** (20)	NR	M	sPCL	t(11;14)	8 prior lines of therapy, including: thalidomide, lenalidomide, bortezomib, carfilzomib, bendamustine, daratumumab and twice high-dose melphalan with AuHSCT	Venetoclax 400 mg daily + CYP3A4 inhibitor + Bortezomib dexamethasone + bendamustine then monotherapy of Venetoclax 400 mg daily + CYP3A4 inhibitor	CR	5 months at time of publication

M, male; F, female; pPCL, primary plasma cell leukemia; sPCL, secondary plasma cell leukemia; Vd, bortezomib+dexamethasone; HyperCVAD-VD, cyclophosphamide, dexamethasone, vincristine, doxorubicin, bortezomib, dexamethasone, methotrexate, cytarabine; CR, complete response; MRD, minimum residual disease; del, deletion; KRd, carfilzomib, lenalidomide, dexamethasone; VdT, bortezomib, dexamethasone, thalidomide; PACE, cyclophosphamide, doxorubicin, etoposide, cisplatin; AuHSCT, autologous hematopoietic stem cell transplantation; VCd, bortezomib, cyclophosphamide, dexamethasone; D-VRd, daratumumab, bortezomib, lenalidomide, dexamethasone; Al-HSCT, allogeneic hematopoietic stem cell transplantation; MM, multiple myeloma; VGPR, very good partial response; HyperCD, hyperfractionated cyclophosphamide and dexamethasone; DVd, daratumumab, bortezomib, dexamethasone; D-KRd, daratumumab, carfilzomib, lenalidomide, dexamethasone; KPD-PAVE, carfilzomib, pomalidomide, dexamethasone, cisplatin, adriamycin, etoposide and venetoclax; KPVd, carfilzomib, pomalidomide, venetoclax, dexamethasone; KCD-PAVE, carfilzomib, cyclophosphamide, dexamethasone, cisplatin, adriamycin, etoposide and venetoclax; N/A, Not applicable; VTCD, bortezomib, thalidomide, cyclophosphamide, dexamethasone; VRCD, bortezomib, lenalidomide, cyclophosphamide, dexamethasone; Rd, lenalidomide; dexamethasone; VRd; bortezomib, lenalidomide, dexamethasone; IRCD, Ixazomib, lenalidomide, cyclophosphamide, dexamethasone; DRd, daratumumab, lenalidomide, dexamethasone; HyperCAD, hyperfractionated cyclophosphamide, doxorubicin and dexamethasone; sCR, stringently defined complete remission, NR, not reported.

The majority of patients [n=6)] achieved sCR/CR (5, 6, 11, 13, 19, 20), VGPR [n=4] (12, 16–18) and PR [n=2] (15), while one patient with secondary PCL and complex karyotype was refractory to the venetoclax combination (14). The treatment was well tolerated in almost all treated patients; grade I thrombocytopenia was reported in three cases, and there was no reported tumor lysis syndrome. The duration of the response was not optimally reported in the majority of cases.

In comparison with the reported literature, this case report offers the latest compilation of the available evidence on the use of venetoclax in patients with PCL. Furthermore, our patient achieved CR for the longest reported durable response in literature thus far, of almost 2 and a half years before he developed lethal COVID-19 pneumonia. The dose of 800 mg was tolerated well in this patient.

The therapeutic potential for the use of the BCL-2 inhibitor, venetoclax, in the treatment of plasma cell neoplasms with t(11;14) is thought to be related to the BCL-2 overexpression in the presence of this translocation (21). Moreover, t(11;14) is linked to a suboptimal response to proteasome inhibitors, thereby rendering the conventional MM combination therapies inadequate (21). Considering that t(11;14) is present in around 50% of patients with PCL, venetoclax is a reasonable molecular alternative for the treatment of PCL (22). This is further supported by the modest evidence presented by the existing literature and the durable response reported herein.

Despite these promising findings, challenges remain in the widespread implementation of venetoclax in the clinical management of PCL. Firstly, the rarity of PCL limits the availability of large-scale clinical trials. This hinders the gathering of the robust and generalizable evidence required to establish standardized treatment guidelines. Additionally, the high cost of venetoclax may pose a barrier to its accessibility, particularly in resource-limited settings.

## Conclusion

PCL has a very poor prognosis despite treatment with myeloma- specific novel agents and/or chemotherapy, including ASCT. Preliminary data for patients with primary or secondary PCL in association with t(11;14) demonstrate a promising response to venetoclax combination therapy with a durable deep response, acceptable toxicity, and improved long- term survival in some cases. Despite these promising findings, the use of venetoclax in PCL is still in its early stages, and larger clinical trials are warranted to establish its efficacy and safety in this patient population. Prospective clinical trials are needed to elucidate the optimal dosage, combination, and duration of treatment, ensuring better representation and generalizability of the findings. Meanwhile, venetoclax may be considered as a therapeutic option in patients with PCL t(11;14).

## Data availability statement

The original contributions presented in the study are included in the article, further inquiries can be directed to the corresponding author.

## Ethics statement

Written informed consent was obtained from the next of kin for the publication of any potentially identifiable images or data included in this article. This case was approved by the Hamad Medical Corporation’s Medical Research Center. MRC number: 04-23-847.

## Author contributions

HE: Writing – review & editing. RG: Writing – review & editing. HEO: Writing – review & editing. MB: Writing – review & editing. HC: Writing – review & editing. RT: Writing – review & editing.

## References

[1] Larrea C Kyle R Rosiñol L Paiva B Engelhardt M Usmani S . Primary plasma cell leukemia: consensus definition by the International Myeloma Working Group according to peripheral blood plasma cell percentage. Blood Cancer J. (2021) 1(12):192. doi: 10.1038/s41408-021-00587-0 PMC8640034 34857730

[2] Chaulagain CP Diacovo M-J Van A Martinez F Fu C-L Jimenez AMJ . Management of primary plasma cell leukemia remains challenging even in the era of novel agents. Clin Med Insights Blood Disord. (2021) 14:2634853521999389. doi: 10.1177/2634853521999389 33716516 PMC7917418

[3] Jung S-H Lee J-J . Update on primary plasma cell leukemia. Blood Res. (2022) 57(S1):62–6. doi: 10.5045/br.2022.2022033 PMC9057670 35483928

[4] Nguyen N Chaudhry S Totiger TM Diaz R Roberts E Montoya S . Combination venetoclax and selinexor effective in relapsed refractory multiple myeloma with translocation t(11;14). NPJ Precis Oncol. (2022) 6(1):73. doi: 10.1038/s41698-022-00315-2 36261486 PMC9581939

[5] Jelinek T Mihalyova J Kascak M Duras J Popkova T Benkova K . Single-agent venetoclax induces MRD-negative response in relapsed primary plasma cell leukemia with t(11;14). Am J Hematol. (2019) 94(1):E35-7. doi: 10.1002/ajh.25331 30370955

[6] Gonsalves WI Buadi FK Kumar SK . Combination therapy incorporating Bcl-2 inhibition with Venetoclax for the treatment of refractory primary plasma cell leukemia with t (11;14). Eur J Hematology. (2017) 100(2):215–7. doi: 10.1111/ejh.12986 29064593

[7] Donk N Lokhorst HM Anderson KC Richardson PG . How I treat plasma cell leukemia. Blood. (2012) 120:2376–89. 10.1182/blood-2012-05-408682 PMC3757364 22837533

[8] Gertz MA Buadi FK . Plasma cell leukemia. Haematologica. (2010) 95:705–7. doi: 10.3324/haematol.2009.021618 PMC2864374 20442443

[9] Szita VR Mikala G Kozma A Fábián J Hardi A Alizadeh H . Targeted venetoclax therapy in t(11;14) multiple myeloma: real world data from seven hungarian centers. Pathol Oncol Res. (2022) 28:1610276. doi: 10.3389/pore.2022.1610276 35295611 PMC8918485

[10] Cotte C Hartley-Brown M . Plasma cell leukemia: Retrospective review of cases at Monter Cancer Center/Northwell Health Cancer Institute, 2014-2019. Curr problems Cancer. (2022) 46(3):100831. doi: 10.1016/j.currproblcancer.2021.100831 35091270

[11] Tang ASO Asnawi AWA Koh AZY Chong SL Liew PK Selvaratnam V . Plasma cell leukemia with successful upfront venetoclax in combination with allogeneic transplantation. Am J Case Rep. (2023) 24:e938868. doi: 10.12659/AJCR.938868 36882990 PMC10009647

[12] Glavey SV Flanagan L Bleach R Kelly C Quinn J Chonghaile TN . Secondary plasma cell leukaemia treated with single agent venetoclax. Br J haematology. (2020) 190(4):e242–5. doi: 10.1111/bjh.16858 32525557

[13] Vo K Guan T Banerjee R Lo M Young R Shah N . Complete response following treatment of plasma cell leukemia with venetoclax and dexamethasone: A case report. J Oncol Pharm Pract. (2022) 28(5):1244–8. doi: 10.1177/10781552221074269 35084252

[14] Zaanona MIA Patel P . Plasma cell leukaemia with t(11;14) not responsive to venetoclax. BMJ Case Rep. (2021) 14(1):e238641. doi: 10.1136/bcr-2020-238641 PMC7839868 33495184

[15] Roy T An JB Doucette K Chappell AM Vesole DH . Venetoclax in upfront induction therapy for primary plasma cell leukemia with t(11;14) or BCL2 expression. Leukemia lymphoma. (2022) 63(3):759–61. doi: 10.1080/10428194.2021.2010065 35076333

[16] Kupsh A Arnall J Voorhees P . A successful case of venetoclax-based therapy in relapsed/refractory secondary plasma cell leukemia. J Oncol Pharm Pract. (2019) 26(5):1274–8. doi: 10.1177/1078155219895072 31865846

[17] Yang Y Fu L-J Chen C-M Hu M-W . Venetoclax in combination with chidamide and dexamethasone in relapsed/refractory primary plasma cell leukemia without t(11;14): A case report. World J Clin cases. (2021) 9(5):1175–83. doi: 10.12998/wjcc.v9.i5.1175 PMC7896656 33644182

[18] Valliani S Ali M Mahmoo O Hinduja S Chen CK Damon L . Efficacy of venetoclax and dexamethasone in refractory igM primary plasma cell leukemia with t(11;14) and TP53 mutation: A case report and literature review. Case Rep Hematol. (2020) 2020:8823877. doi: 10.1155/2020/8823877 33425404 PMC7781713

[19] Nalghranyan S Singh AP Schinke C . The combination of venetoclax, daratumumab and dexamethasone for the treatment of refractory primary plasma cell leukemia. Am J Hematol. (2020) 95(2):E34–5. doi: 10.1002/ajh.25676 31709578

[20] Mikala G Cegledi A Csacsovszki O Peto M Szemlaky Z Udvardy M . PB2233 - Minimal residual disease negativity after molecularly targeted venetoclax therapy of secondary plasma cell leukemia with translocation t(11;14). 23rd Congress of the European Hematology Association. (2018) PB2233.

[21] Hui S Huayuan Z Jianyong L . Progress of BCL-2 inhibitors in chronic lymphocytic leukemia. J Clin Hematol. (2022) 35(1):77–81. doi: 10.13201/j.issn.1004-2806.2022.01.015

[22] Pan D Richter J . Where we stand with precision therapeutics in myeloma: prosperity, promises, and pipedreams. Front Oncol. (2021) 11:819127. doi: 10.3389/fonc.2021.819127 35127532 PMC8811139

